# Characteristics of Indonesian Stingless Bee Propolis and Study of Metabolomic Properties Based on Region and Species

**DOI:** 10.3390/molecules29174037

**Published:** 2024-08-26

**Authors:** Diah Kartika Pratami, Muhamad Sahlan, Asep Bayu, Masteria Yunovilsa Putra, Baharudin Ibrahim, Siswadi, Rahmatul Qodriah, Abdul Mun’im

**Affiliations:** 1 Faculty of Pharmacy, Universitas Indonesia, Cluster of Health Sciences Building, Depok 16424, West Java, Indonesia; d.kartika@univpancasila.ac.id; 2 National Metabolomics Collaborative Research Center, Faculty of Pharmacy, Universitas Indonesia, Kampus UI, Depok 16424, West Java, Indonesia; asep044@brin.go.id (A.B.); mast001@brin.go.id (M.Y.P.); 3 Center for Study of Natural Product for Degenerative Disease, Faculty of Pharmacy, Pancasila University, South Jakarta 12640, DKI Jakarta, Indonesia; rahmatulqodriyah@univpancasila.ac.id; 4 Department of Chemical Engineering, Faculty of Engineering, Universitas Indonesia, Depok 16425, West Java, Indonesia; sahlan@che.ui.ac.id; 5 Research Center for Biomedical Engineering, Faculty of Engineering, Universitas Indonesia, Depok 16425, West Java, Indonesia; 6 Research Center for Vaccine and Drug, National Research and Innovation Agency (BRIN), Bogor 16911, West Java, Indonesia; 7 Faculty of Pharmacy, Universiti Malaya, Kuala Lumpur 50603, Malaysia; baharudin.ibrahim@um.edu.my; 8 Research Center for Pharmaceutical Ingredients and Traditional Medicine, National Research and Innovation Agency, Bogor 16911, West Java, Indonesia; siswadi@brin.go.id

**Keywords:** Indonesian propolis, *Tetragonula laeviceps*, *Heterotrigona itama*, marker compounds, metabolomics

## Abstract

The chemical compounds found in propolis vary according to plant sources, species, and geographical regions. To date, Indonesian propolis has not yet become standardized in terms of its chemical constituents. Thus, this study aimed to identify the presence of marker compounds and determine whether different classes of Indonesian propolis exist. In this study, yields, total polyphenol content (TPC), total flavonoid content (TFC), and antioxidants were measured. Identification of chemical compounds was carried out with Fourier-transform infrared (FTIR) spectroscopy and liquid chromatography-tandem mass spectrometry (LC-MS/MS). Metaboanalyst 6.0 was employed in conducting principal component analysis (PCA) and partial least squares discriminant analysis (PLS-DA) using the results of the FTIR and LC-MS/MS. The propolis with the highest TFC, TPC, and antioxidant activity was *Geniotrigona thoracica* from North Sumatra. The results of propolis compound mapping based on region with discriminant analysis revealed that types of propolis from Java have similar characteristics. Then, based on species, the types of propolis from *Tetragonula laeviceps* and *Heterotrigona itama* have special characteristics; the samples from these species can be grouped according to similar characteristics. In conclusion, 10 potential marker compounds were identified in Indonesian propolis, enabling regional and species-specific varieties of Indonesian propolis to be classified based on chemical composition mapping.

## 1. Introduction

Propolis, a natural resin produced by bees, is used in traditional medicine and in food, beverages, and supplements to improve health and prevent infection, inflammation, heart disease, and cancer [1,2]. Propolis also has a range of applications across various industries due to its unique properties. Here are some notable uses of propolis in different sectors: cosmetics and personal care, pharmaceuticals, agriculture, and veterinary medicine [3,4,5,6]. The array of chemical compounds contained in propolis depends on plant sources, geographical location, environmental conditions, and the species of bee [7,8,9,10]. These factors result in a wide diversity in the chemical compounds found in propolis across regions, the most common being polyphenols and flavonoids [4,5,6]. Indonesia’s high biodiversity is due to its position between two continental shelves, with the Wallace Line separating Asian and Australian faunal regions through the Lombok Strait between Bali and Lombok, and between Borneo and Sulawesi [11].

The metabolomic study investigated the metabolites present in propolis with a comprehensive analysis. The study identified and categorized a diverse range of propolis metabolites [12,13]. Secondly, the study investigated the potential health benefits of the identified metabolites, such as antibacterial [14,15,16], antioxidant [15,17,18,19], anti-inflammatory [20,21], and anti-cancer activities [22,23]. Last, it examined how the metabolomic profile of propolis varies with its region [24,25,26] and botanical sources [27]. The propolis extract in metabolomic research was identified using different analytical techniques, such as liquid chromatography-mass spectrometry (LC-MS) and gas chromatography-mass spectrometry (GC-MS), to identify and measure the amounts of metabolites [24,26,28,29,30,31]. Nuclear Magnetic Resonance (NMR) spectroscopy is also used to determine the structure of metabolites [12,25,32,33,34,35]. Fourier-transform infrared (FTIR) can be used to evaluate the functional group of the propolis compound [17]. The data analysis of the metabolomic study employs bioinformatics techniques to scrutinize the data, discern metabolites, and elucidate the findings. The bioactivity assays involve the performance of in vitro and in vivo studies to evaluate the biological activities of the discovered metabolites.

According to the current literature, a key subject of interest is the profile of the chemical compounds in propolis. So, the point of this study was to find out if there are different types of Indonesian propolis based on region and species, as well as to see if there are different classes of Indonesian propolis. The establishment of stingless bee propolis will facilitate testing the quality, support marketing, and aid in increasing the production of Indonesian propolis.

## 2. Results

### 2.1. Bee Species

**Figure 1:**
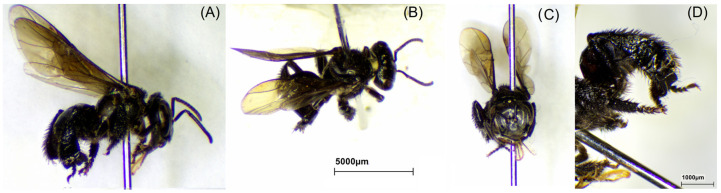
Morphology of *H. itama*. (**A**) Full body; (**B**) dorsal view; (**C**) front view; (**D**) hind tibia.

**Figure 2:**
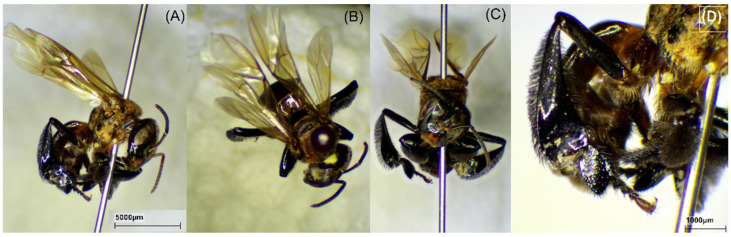
Morphology of *G. thoracica*. (**A**) Full body; (**B**) dorsal view; (**C**) front view; (**D**) hind tibia.

**Figure 3:**
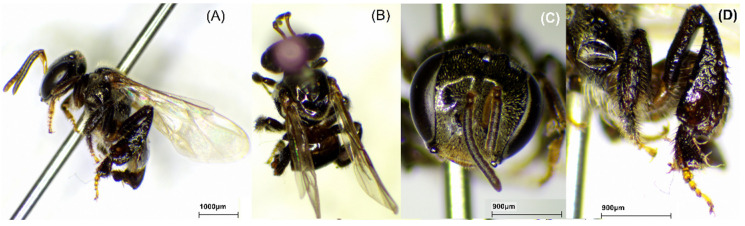
Morphology of *T. laeviceps*. (**A**) Full body; (**B**) dorsal view; (**C**) front view; (**D**) hind tibia.

**Figure 4:**
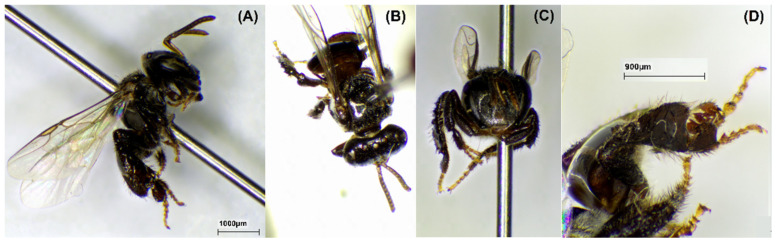
Morphology of *T. biroi*. (**A**) Full body; (**B**) dorsal view; (**C**) front view; (**D**) hind tibia.

The bee species was identified to confirm the origin of propolis. The morphology of stingless bees *H. itama*, *G. thoracica*, *T. laeviceps*, and *T. biroi* is presented in Figure 1, Figure 2, Figure 3 and Figure 4.

### 2.2. Total Phenolic Content (TPC) Results

The calculation of the gallic acid calibration curve resulted in the following equation: y = 0.0074x + 0.0376 (R^2^ = 0.999). After the sample absorbance data were obtained, the original concentration of the sample in ppm could be calculated using the calibration curve equation. Subsequently, the conversion was carried out for the TPC of extracts of stingless bee propolis (EEP) from various Indonesian regions (Table 1). The TPC ranged from 32.99 ± 0.66 to 374.20 ± 0.51 mg of gallic acid equivalents (GAE)/g. Propolis from *G. thoracica* from Pematang Siantar, Sumatra (sample code Sum_GT4), had the highest concentration of TPC, whereas the lowest total value of polyphenol content was found in propolis from Barito Kuala, Kalimantan (sample code Kal_TL5).

### 2.3. Total Flavonoid Content (TFC) Results

The calculation of the quercetin calibration curve resulted in the following equation: y = 0.0069x − 0.0687 (R^2^ = 0.9974). After the absorbance data were obtained from the sample, the original concentration of the sample in ppm could be calculated using the calibration curve equation. According to the conversion that was subsequently carried out, the TFC values of EEP for various islands are shown in Table 1. The TFC of Indonesian stingless bee propolis ranged from 20.66 ± 4.08 to 194.18 ± 0.98 mg QE/g. Propolis from Pematang Siantar, Sumatra (sample code Sum_GT4), had the highest TFC. Meanwhile, the lowest overall value of flavonoid content was found in propolis from Barito Kuala, Kalimantan (sample code Kal_TL5).

### 2.4. Antioxidant Activity Results

The results for the antioxidant activity of the EEP using the DPPH method are shown in Table 2, showing the lowest IC_50_ for *T. laeviceps* propolis from Kendal, Central Java, at 118.1 ± 2362 ppm (moderate). The highest IC_50_ activity was 11.06 ± 0.55 ppm (very strong) in *G. thoracica* propolis samples from Pematang Siantar, North Sumatra.

The antioxidant activity of EEP determined using the ABTS method showed that IC_50_ ranged from 17.29 ± 0.34 to 117.21 ± 10.76 ppm. The EEP *G. thoracica* from Pematang Siantar, North Sumatra, had the strongest activity. The samples with lowest activity were propolis samples *T. biroi* from Batang, Central Java. The classification of antioxidant strength in Table 2 is based on previous research, which classifies antioxidant strength as very strong (<50 ppm), strong (50–100 ppm), moderate (101–250 ppm), weak (250–500 ppm), and inactive (>500 ppm) [36].

### 2.5. Chemometric Analysis of FTIR Spectroscopies

**Figure 5:**
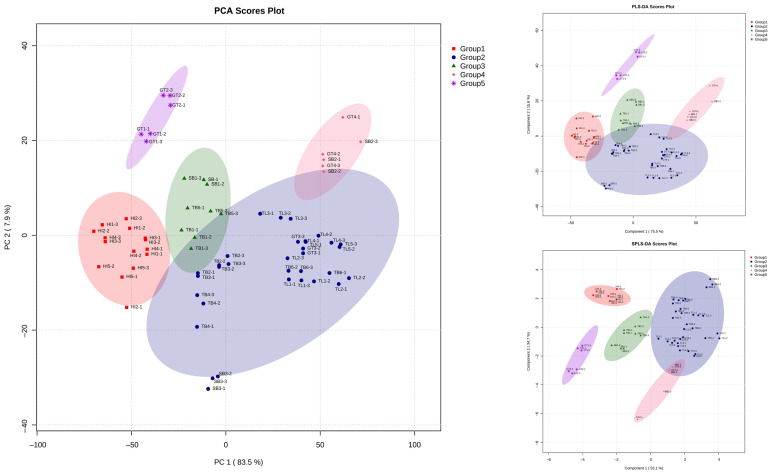
Chemometric analysis of propolis spectra from FTIR.

The results of the chemometric analysis of the principal component analysis (PCA), partial least squares discriminant analysis (PLS-DA), and sparse partial least squares discriminant analysis (sPLS-DA) of 23 samples of propolis using Metaboanalyst 6.0 are shown in Figure 5. The score plots of PCA, PLS-DA, and SPLS-DA show consistent clustering and discriminant results. From the score plot of chemometrics analysis, the red area of Group 1 consists of all samples from the same species, *H. itama*. Its regions were Kendal (Java), Magelang (Java), Banjar Baru (Kalimantan), Kutai Kartanegara (Kalimantan), and Kampar (Sumatra). Group 2 with the purple color is *G. thoracica* from Kutai Kartanegara (Kalimantan) and Lebong (Sumatra). The blue area, Group 3, with its blue color, consists of eleven samples from species *T. biroi*, *T. laeviceps*, *T. drescheri*, and *G. thoracica*. This group included all *T. laeviceps* propolis samples. The region of samples in this group was Tanah Laut (Kalimantan), Batang (Java), Pandeglang (Java), Magelang (Java), Hulu Sungai Tengah (Kalimantan), and Barito Kuala (Kalimantan). The *T. biroi* propolis in Group 3 was from Tanah Laut (Kalimantan), Batang (Java), Bogor (Java), and Magelang (Java). The last species in this group comprised propolis (*T. drescheri*) from Batang (Java) and *G. thoracica* from Hulu Sungai Tengah (Kalimantan). The green area (Group 4) consists of *T. biroi*, *T. clypearis* (Nusa Tenggara), and *T. biroi* (Kalimantan). The last area, with a pink color (Group 5), consists of *T. clypearis* (Nusa Tenggara) and *G. thoracica* (Pematang Siantar). Table 3 describes each member in every group.

**Figure 6:**
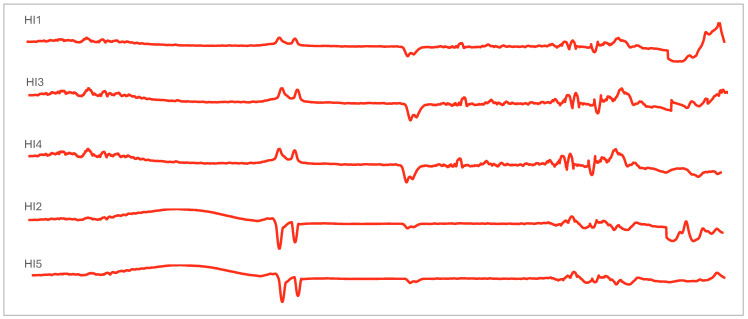
FTIR stingless bee propolis spectra of Group 1.

**Figure 7:**
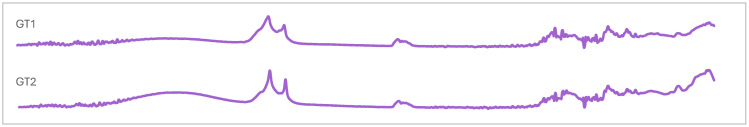
FTIR stingless bee propolis spectra of Group 2.

**Figure 8:**
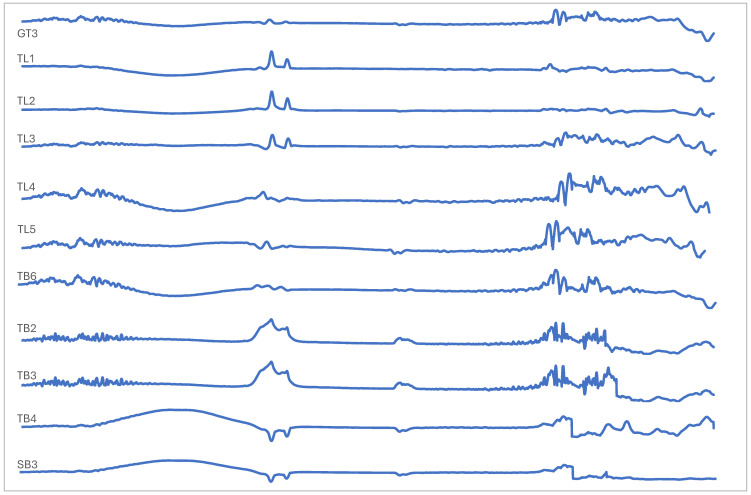
FTIR stingless bee propolis spectra of Group 3.

**Figure 9:**
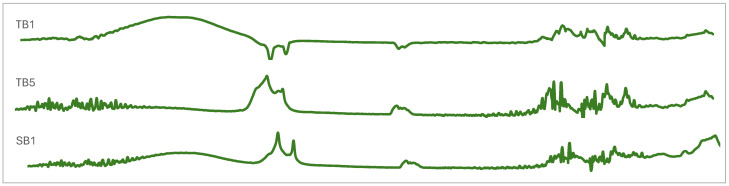
FTIR stingless bee propolis spectra of Group 4.

**Figure 10:**
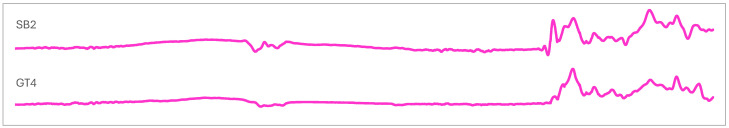
FTIR stingless bee propolis spectra of Group 5.

The propolis spectra from FTIR per group are shown in Figure 6, Figure 7, Figure 8, Figure 9 and Figure 10. They demonstrate that stingless bee propolis from the various species and regions represented in the study revealed a spectrum with different vibrations, characterized by transmittance in wavenumber. We can attribute this observation to the similar composition of certain species and inter-island propolis compounds. Table 4 displays the combined readings of the propolis vibrational bands and compounds identified from the FTIR results.

FTIR analysis can be used in metabolomics studies and is considered an efficient and economical analysis technique for classifying bioactive metabolic profiles, but it has the limitation of not being able to identify marker molecules from the sample. We conducted LC-MS/MS analysis of propolis samples and considered the most suitable analytical technique for characterizing propolis bioactive metabolic profiles from those species, especially *H. itama* and *T. laeviceps* [37].

### 2.6. LC-MS/MS Analysis Result

**Figure 11:**
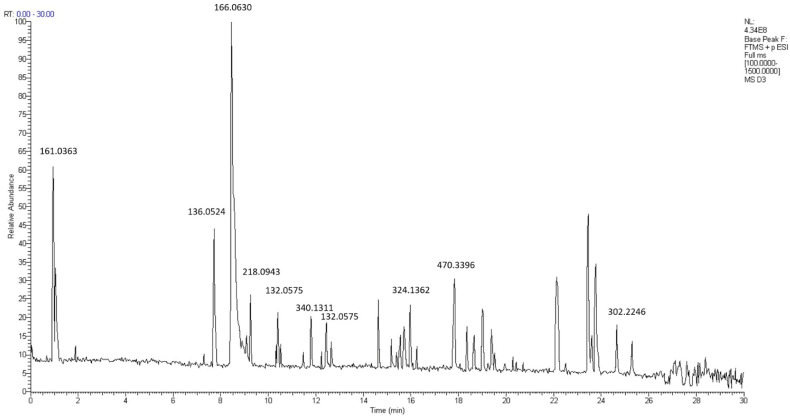
LC-MS/MS spectra of propolis Sum_GT4.

The study analyzed the bioactive compounds of 11 samples of propolis (all *H. itama* and *T. laeviceps*) using LC-MS. The spectra of propolis *G. thoracica* from Pematang Siantar (sample code Sum_GT4), which has the highest phenolic and flavonoid content and also the best activity as an antioxidant, can be seen in Figure 11 and Table 5.

### 2.7. Chemometric Analysis of LC-MS/MS

This study employed LC-MS/MS to identify the chemical compounds of propolis samples. The chemometric analysis of the FTIR sample shown in Figure 5 shows that *H. itama* from Java (Kendal, Magelang), Kalimantan (Banjar Baru, Kutai Kartanegara), and Sumatra (Kampar) have a near score plot, so they cluster in Group 1. Propolis samples from *T. laeviceps* from Java (Pandeglang, Batang, and Magelang) and Kalimantan (Hulu Sungai Tengah and Barito Kuala) also have a near score plot, so they cluster in Group 3.

**Figure 12:**
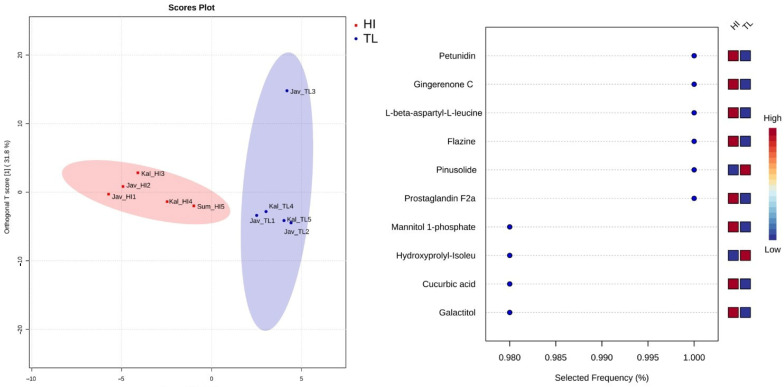
Score plot S-PLSDA for propolis *H. itama* vs. *T. laeviceps* and molecule markers.

The chemometric analysis with MetaboAnalyst 6.0 software showed good discriminant analysis and metabolomic compounds. The results of OPLS-DA are shown in Figure 12. The identification of similar compounds that occurred in those species groups was carried out as a means of searching for marker compounds. We identified 15 distinct compounds in Indonesian propolis overall. This observation suggests that these compounds have the potential to act as marker compounds. Table 6 presents a list of these compounds.

## 3. Discussion

### 3.1. TPC and TFC

The values of TPC and TFC of propolis samples from *G. thoracica, H. itama, T. biroi, T. laeviceps,* and another stingless bee were varied. According to the study results, the TPC and TFC of this study were lower than previous research on bees of the genus *Tetragonula spp.* from Luwu, South Sulawesi, with a range of 269.57 ± 20.37–426.91 ± 61.08 mg GAE/g and 211.08 ± 10.14 to 324.43 ± 11.84 mg QE/g [38]. However, the results of this study are in accordance with the TPC results of propolis samples from Anatolian Turkey, with varying values of 16.37–125.83 mg GAE/g [39]. This study range is equivalent to Polish propolis, with a TFC between 35.64 and 62.04 QE/g [40]. In terms of TPC value, this study is slightly higher than the reported samples of Malaysian propolis species *H. fimbriata, T. apicalis,* and *T. binghami*, with TPC values of 13.21 ± 0.26, 7.60 ± 0.13, and 10.11 ± 0.19 mg RE/g [41]. In this study, the TFC value obtained was higher than the results reported by Fikri et al. in 2019, using Banten, South Sulawesi, and South Kalimantan propolis; the flavonoid range was obtained as 0.76 ± 0.26–3.39 ± 1.08 mg QE/g [42].

Variations in TPC values in this study show that different results can occur in the same bee species, and differences in the region of origin of propolis also show variations in TPC content. The TPC and TFC vary greatly, which can occur due to the type of vegetation around the colony, bee species, origin, solvent, and/or extraction method used [42]. This difference in variation can be used as a standardization reference for the TPC and TFC content of stingless bee propolis in Indonesia, because it has been taken from several regional points and samples of different species. The challenge is to obtain globally agreed-upon standards for use in the TPC testing of stingless bee propolis internationally [43]. Phenolic compounds are components related to biological activity whose levels can be used as standards in propolis extract content, for example, phenolic compounds (chlorogenic acid, caffeic acid, isochlorogenic acid, caffeic acid phenethyl ester, and artepillin C) and flavonoids (myrisetin, quercetin, kaempferol, apigenin, pinocembrin, and galangin), which are used as standards in Brazilian and Chinese propolis [44].

These values are also associated with extraction techniques, solvents, and extraction times [45]. Previous research has shown that obtaining a high TPC requires the propolis sample to be extracted using a robust extraction method, such as ultrasound-assisted extraction (UAE), with an extraction time of less than 0.5 h [45]. In contrast, achieving a high TFC requires that samples should be extracted using conventional methods of extraction, such as maceration, which typically involves an extraction time of more than 0.5 h [45]. The optimal solvent for obtaining propolis extractions with high TFC and TPC is aqueous ethanol [45].

### 3.2. Antioxidant Activity

The IC_50_ value for reducing DPPH radicals from EEP in this study was the same range as propolis from several countries, such as Bolivian propolis with 4.54–48.27 µg/mL [46]; Propolis Stingless Bees (Meliponinae) Tocantins, Brazil, at 29.81 ± 2.49–50.23 ± 1.60 µg/mL [47]; and Chinese propolis at 15.49 ± 70.59–28.69 ± 71.52 µg/mL [48].

The antioxidant activity with ABTS methods was in line with previous research on stingless bee propolis from Malaysia, which showed the highest antioxidant activity found in the *G. thoracica* species, with a value of 64.98 ppm [49].

Research on propolis extracts from Malaysia, the species *Tetrigona apicalis, H. itama*, and *G. thoracica*, shows a strong correlation between total phenolic and flavonoid levels and antioxidant activity [49]. The biological activity of propolis is chiefly due to the presence of phenolic and flavonoid compounds, as well as variations in the quantities of these components [50]. The polyphenol and flavonoid content of propolis has been found to have a linear correlation with its antioxidant activity [38,49], with higher levels of polyphenols and flavonoids associated with increased antioxidant activity. The study shows a linear relationship between antioxidant activity and TPC and TFC.

The antioxidant activity of phenolic compounds is associated with their ability to eliminate free radicals by donating hydrogen atoms, electrons, or metal cations; this interaction capacity with free radicals is due to their structure (mainly due to the number and position of hydroxyl groups and the nature of substitution in the aromatic ring) but is also based on the binding of the compound with organic acids and sugars [51].

### 3.3. Vibrational and Absorption Spectroscopies of Indonesian Propolis

In all samples, the classes of compounds identified in propolis include alcohols, phenols, carboxylic acids, alkenes, and aromatic rings, suggesting that all types of Indonesian propolis contain alkyl and aromatic compounds characterized by amine, ester, alkyl, and hydroxyl functional groups [48,49,50,51,52,53]. In addition, these results further confirm the presence of aromatic acids, flavonoids, and polyphenol acids in propolis [54,55,56,57].

FTIR analysis can also be an effective method for determining the presence of various functional groups in a compound or molecule [58]. The main common compounds found in propolis include phenolic acid or its ester (caffeic acid phenylethylester/CAPE), flavonoids (flavones, flavanones, flavonols, dihiroflavonols, and chalcones), terpenes, aldehydes and aromatic alcohols, fatty acid, stilbene, and β-steroids [59]. In addition, aliphatic acids and their esters were also found, as well as aromatic acids and their esters and amino acids. The amino acids contained in propolis, namely arginine and proline, are among the most abundant [60]. There is also research that identifies propolis based on FTIR analysis of the presence of lipid compounds (2910–2845 cm^−1^), monoterpenes (1592, 1114, 1022, 972 cm^−1^), sesquiterpenes (1472 cm^−1^), and sucrose (1122 cm^−1^) [61].

In this case, a discriminant analysis method using Metaboanalyst 6.0 was applied to determine the classification based on the characteristics of propolis across different species and geographical regions. The discriminant analysis method in this study was carried out by observing the distribution area of the entire spectrum, which spanned 4000–500 cm^−1^. Mapping from the FTIR test using the discriminant analysis method showed similar characteristics for propolis compounds divided into five groups. According to the results, the propolis groups from the *H. itama* sample displayed similar characteristics to those groups. The *H. itama* bee collects nectar, pollen, and resin to produce honey, bee bread, and propolis from various types of plants near the colony. Previous research has found that this species of bee prefers to visit certain types of vegetation [62]. *H. itama* prefers to forage in areas closer to the nesting site, where diverse food sources are found. The marked bees of *H. itama* prefer to forage on various resources available within a 500 m radius of the house yard. The resin value of 34.73% was material that was brought back to the hive by foragers [63].

This also happened in the propolis group from *T. laeviceps,* which has a near score plot. The prevalence of resin from *Pinus sp*. in the nests of *T. laeviceps* was found to be a primary source in the production of propolis [64]. This preference for *Pinus sp*. resin might explain why *T. laeviceps* frequently visit places that offer both resin and pollen as their food source.

On the other hand, the third group of FTIR maps consists of mixed propolis samples. They are not grouped by the same species. This may be caused by the source of vegetation or geographical origin. Propolis T*. biroi, T. laeviceps*, and *T. drescheri* from Batang have a near score plot of PLSDA. This phenomenon also occurred in propolis samples belonging to T*. biroi* and *T. laeviceps* from Magelang, as well as *T. laeviceps* and G*. thoracica* from Hulu Sungai Tengah. This phenomenon can be evaluated by the fact that *T. biroi*, *G. thoracica, T. clypearis*, and *T. drescheri* exhibit preferences in foraging behavior around their nests. These preferences are influenced by factors such as the location of food sources and the types of plants that can provide resin for producing propolis.

### 3.4. Propolis Chemical Compounds and Potential Markers

The chemical compounds in propolis include a rich variety of phenolic molecules. Often referred to as polyphenols, these compounds possess at least one aromatic ring and one or more hydroxyl functional groups. Flavonoids, which represent the most abundant group of phenolic compounds, have structures based on a C6-C3-C6 skeleton and are subdivided into several classes that differ in the oxidation state of the central heterocyclic ring. The classes are comprised of flavones, flavonols, flavanones, chalcones, isoflavonoids, flavanols (catechins and tannins), and anthocyanidins. Non-flavonoids include phenolic acids, simple phenols, coumarins, xanthones, lignins, lignans, and stilbenes. Phenolic acids can be divided into benzoic acid derivatives (based on a C6-C1 skeleton) and cinnamic acid derivatives (based on a C6-C3 skeleton). The variability of chemical composition in propolis can be attributed to large numbers of phenolics from different classes, such as glycoside phenolic compounds, and highlights the challenges associated with the analysis of propolis samples [43].

In stingless bee propolis from sources across the globe, investigations of chemical composition to date have identified many polyphenolic compounds. Based on their molecular structure, these compounds can be divided into flavonoids and nonflavonoids. The flavonoid compounds include flavone (chrysin), flavonol (galangin), and flavanone (pinocembrin). The nonflavonoid compounds are phenolic acids, which can be further subdivided into benzoic acid derivatives, such as gallic acid and protocatechuic acid, and cinnamic acid derivatives, such as caffeic acid, p-coumaric acid, and ferulic acid [65].

Based on analysis using LC-MS/MS and MetaboAnalyst software, 10 propolis compounds were identified as potential markers for stingless bees from *H. itama* and *T. laeviceps* propolis. In addition to the identification of those species classes based on the results of compound mapping from LC-MS/MS using PLSDA, they contain similar chemical compounds with similar characteristics.

The 10 molecular marker compounds of *H. itama* propolis identified in this study are petunidin, gingerone C, L-β-aspartyl-L-leucine, flazine, prostaglandine F2a, mannitol 1-phosphate, cucurbic acid, and galacticol. On the other hand, pinusolide and hydroxyprolyl-Isoleucine were identified as high in *T. laeviceps* propolis. Petunidin, a blue antochianidine flavonoid, was found in the bee pollen of *Echium plantagineum* [66] and *Fuchsia excorticata* [67]. Gingerenone C is a polyphenol found in *Zingiber officinalle* [68]. Flazine is an indole alkalloid compound from *Brucea javanica* [69]. Cucurbic acid is a natural compound found in *Solanum tuberosum, Solanum lycopersicum*, *Peponapis limitaris, Cucurbita moschata,* and other plants in the Cucurbitaceae [70]. Mannitol and gallacticol are sugar alcohols that can be found in bee products [71]. L-β-aspartyl-L-leucine and hydroxyprolyl-Isoleucine are amino acid derivatives that are needed for the development of bee colonies. Pinusolide is a diterpene lactone, a metabolite of *Agathis macrophylla*, *Pinus armandii,* and other Pinus species. This compound is high in *T. laeviceps* propolis, because it supplies resin and pollen as a bee food source [72].

Different kinds of plants have different resins with different chemical compositions, and even different individuals within the same species might have different resins. Even more widespread is resin utilization in stingless bees; several species of stingless bees gather large quantities of resin, which they then utilize to bolster various parts of colony function [24,25,26].

## 4. Materials and Methods

### 4.1. Samples

**Figure 13:**
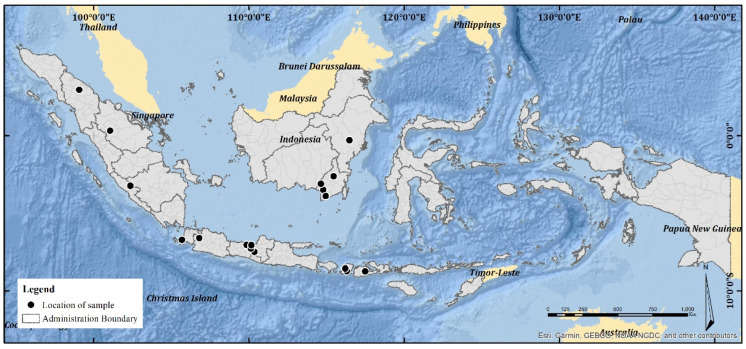
Distribution of samples.

We studied a total of 23 raw propolis samples collected from 17 cities in Indonesia (Figure 13). The species were *H. itama* (HI), *T. laeviceps* (TL), *T. biroi* (TB), *G. thoracica* (GT), and others stingless bees, *T. clypearis* and *T. drescheri,* with a code of SB. The curator of the Zoologicum Museum, University of Indonesia (UIMZ), Biota Collection Room (RKBUI), Department of Biology, FMIPA, Universitas Indonesia, carried out the identification of bee species. We examined the morphology of bee species using the digital microscope DeltaPix DPX M12000 (DeltaPix, Smoerum, Denmark). Table 7 provides the list of samples.

### 4.2. Sample Preparation

The raw materials for propolis were obtained after harvesting honey by squeezing or sucking it from the beehives. After harvesting the honey, the bee bread, bee pollen, and dead bees were separated from the beehive. Then, propolis was frozen at −18–20 °C for a day until it reached a hard texture or was no longer soft. Frozen propolis was then crushed using a chopper to obtain fine flakes. Propolis in the form of fine granules could be used for extraction.

### 4.3. Propolis Extraction

A total of 50 g of fine propolis was macerated with 250 mL of 96% ethanol. The resultant mixture was stirred at 355 rpm for 8 h. The extract was subsequently left overnight and then filtered using a Buchner funnel (Deschem, Changshu, China) lined with filter paper with a pore diameter of 10 µm. The filtrate was designated as EEP 96% (ethanol extract propolis 96%), while the cake was designated as resin. After filtering, EEP 96% was diluted with distilled water until a concentration of 70% ethanol was reached. The diluted EEP was incubated for 30 min at 50 °C in a water bath. The solution was then stored in the refrigerator overnight at 0–5 °C. The wax precipitate was filtered using a Buchner funnel to obtain a filtrate, which was then concentrated using a rotary evaporator to obtain a thick ethanol extract of propolis. For extracts that were still not thick, the extract was dried in a food dehydrator (FDH) at a temperature of 50 °C for 24 h. The ethanol extract of propolis (EEP) obtained was stored in the refrigerator until used for testing [38].

### 4.4. Total Phenolic Content (TPC) Measurements

Quantitative tests of TPC were carried out using the 96-well microplate method specified in previous research [38]. A standard of gallic acid (50 mg) was dissolved in methanol to obtain a concentration of 1000 ppm, which was then diluted with water to obtain series concentrations of 12.5–250 ppm.

A 25 μL sample of EEP or gallic acid standard, added with 100 μL of Folin–Ciocalteu reagent (25%), and then incubated for 4 min. Then, we added 75 μL of 1 M Na_2_CO_3_ solution and shook for 60 s, before incubating for 2 h at room temperature. Absorbance was measured at λ 765 nm using a 96-well microplate reader spectrophotometer (Thermo Scientific Multiskan GO, Waltham, MA, USA). The absorbance standard solution of gallic acid was measured at each concentration, and then the calibration curve equation was calculated to obtain y = a + bx and the R^2^ value, where y is the absorbance of gallic acid or sample and x is phenolic compound levels expressed in GAE (gallic acid equivalent). The measurements were carried out in triplicate. The total phenolic value was expressed in mg GAE/g extract.

### 4.5. Total Flavonoid Content (TFC) Measurements

Quantitative tests of TFC were carried out using the AlCl_3_ method specified in previous research [38]. A standard solution of quercetin (10 mg) was dissolved in methanol to obtain a concentration of 1000 ppm, which was then diluted with water to obtain series concentrations of 12.5–250 ppm. A 20 μL sample of EEP or quercetin standard at different concentrations was pipetted and then mixed with 10%AlCl_3_ (20 µL), 1M CH_3_COOK (20 µL), and water (140 mL) and shaken for 60 s. The mixture was left to incubate for 30 min at room temperature. The absorbance of the propolis sample was subsequently measured at a wavelength of 415 nm using a 96-well microplate reader spectrophotometer (Thermo Scientific Multiskan GO, USA). The measurements were carried out in triplicate.

The absorbance standard solution of quercetin was measured at each concentration, and then the calibration curve equation was calculated to obtain y = a + bx and the R^2^ value, where y is the absorbance of quercetin or sample and x is flavonoid compound levels expressed in mg QE/g extract.

### 4.6. Antioxidant Activity with DPPH Assay

Antioxidant activity was conducted using the previous method, as specified in Table 8 and a 96-well microplate reader spectrophotometer (Thermo Scientific Multiskan GO, USA). The percentage of DPPH inhibition was calculated using the formula in Equation (1). Antioxidant activity is expressed by the IC_50_ obtained from the calibration curve.

(1) $$Inhibition\;(\%)=1-(\frac{Asample}{Acontrol})\times 100$$

### 4.7. Antioxidant Activity with ABTS Assay

ABTS•+ were generated according to the previous method of Wołosiak et al. [73], with slight modification by mixing equal volumes of substrate solution (2,2′-azino-bis(3-ethylbenzothiazoline-6-sulfonic acid, 7 mM) and oxidant (potassium persulfate, 2.45 mM); the reaction was carried out in the dark for 16 h. After this time, the radical solution obtained was diluted with ethanol. A total of 50 µL of EEP solutions (with a series concentration of 50–625 ppm) was collected in test tubes, and then 50 µL of the radical solution was added (Table 9). The reaction was performed for a standard time (6 min), measured at 734 nm.

### 4.8. Fourier-Transform Infrared Analysis

Unprocessed raw propolis samples were analyzed with a Fourier-transform infrared (FTIR) alpha spectrophotometer (Bruker Optik, Ettlingen, Germany). Stingless bee propolis samples were placed on the ATR crystal. All spectra were acquired between 4000–400 cm^−1^, with a resolution of 4 cm^−1^ and a signal speed of 32 waves/minute. Each sample was tested in triplicate. Data point tables (DPTs) were used to store IR spectra in ASCII format, which were converted to Microsoft Excel tables in the form of (.csv) files. The spectrum data used was total transmittance information [74].

### 4.9. LC-MS/MS Analysis

The LC-MS/MS analyses were performed using UHPLC Vanquish Tandem Q Exactive Plus Orbitrap HRMS (Thermo Scientific, Waltham, MA, USA) in the Advanced Research Laboratory, IPB University. The signal read on the LC-MS/MS device was analyzed using Thermo Scientific Xcalibur 4.2 software to obtain a chromatogram. Data generated by the XCalibur software was further processed using Compound Discoverer 3.2 software. The column was Accucore C18, 100 × 2.1 mm, 1.5 µm (Thermo Scientific Waltham, MA, USA), the temperature was set at 30 °C, and the flow rate was 0.2 mL/min. The analyses were performed using a linear gradient solvent system consisting of A:B (0.1% formic acid in H_2_O:0.1% formic acid in acetonitrile) as follows: t = 0–1 min 5% B; t = 1–25 min 5–95% B; t = 25–28 min 95% B; t = 28–30 min 5% B. EEP samples were filtered using a 0.2 µm PTFE membrane filter (Merck KGaA, Darmstadt, Germany). The injection volume was 5.0 µL, and MSE positive ionization ESI electrospray had an acquisition range of 100–1500 m/z [38]. The spectra of each sample were saved as (.raw) or (.MzML). The data acquisition and interpretation were processed using mzcloud, chemspider, and the Human Metabolome Database (HMDB).

### 4.10. Chemometric Analysis

Chemometric analysis with Metaboanalyst 6.0 software was conducted by employing principal component analysis (PCA), partial least squares discriminant analysis (PLS-DA), and sparse partial least squares discriminant analysis (sPLS-DA) to map the chemical compounds in stingless bee Indonesian propolis. The FTIR data on the (.csv) and LC-MS/MS data (.mzML) files were statistically analyzed. The data from 23 samples were preprocessed before chemometric analysis. After obtaining the normalized data, PCA, PLS-DA, and sPLS-DA were conducted for integrative data analysis [75].

## 5. Conclusions

The conclusions drawn in this study are based on the analysis of propolis compounds using FTIR and LC-MS/MS. Our findings are in line with the reports of previous scholars from different sources. The metabolite compounds were correlated with bee species and phytogeographic sources. The results of propolis compound mapping from the FTIR test using discriminant analysis demonstrated that the types of propolis from Java have similar characteristics. Then, based on species, the types of propolis from *T. laeviceps* and *H. itama* have special characteristics; the samples from each species can be grouped according to similar characteristics. Using LC-MS/MS analysis, we identified 10 propolis compounds as potential markers for Indonesian propolis from *H. itama* and *T. laeviceps.* In conclusion, a metabolomic study was conducted on Indonesian stingless bee propolis, enabling regional and species-specific varieties of propolis to be classified based on chemical composition mapping.

## Figures and Tables

**Table 1:** Results of total polyphenol content (TPC) and flavonoid content (TFC).

Sample name	Bee Species	Region	TPC ± SD (mg GAE/g)	TFC ± SD (mg QE/g)
Jav_HI1	*H. itama*	Magelang	61.42 ± 5.13	63.74 ± 7.47
Jav_HI2	*H. itama*	Kendal	69.72 ± 4.89	52.22 ± 5.27
Kal_HI3	*H. itama*	Banjarbaru	278.78 ± 15.79	74.40 ± 3.63
Kal_HI4	*H. itama*	Kutai Kartanegara	208.8 ± 10.12	112.8 ± 5.24
Sum_HI5	*H. itama*	Kampar	67.56 ± 2.84	70.11 ± 2.73
NT_TB1	*T. biroi*	Sumbawa	336.96 ± 0.64	111.40 ± 0.81
Kal_TB2	*T. biroi*	Tanah Laut	97.61 ± 1.96	59.49 ± 3.22
Jav_TB3	*T. biroi*	Batang	83.66 ± 2.21	60.74 ± 3.95
Jav_TB4	*T. biroi*	Bogor	114.48 ± 5.35	48.5 ± 0.57
Kal_TB5	*T. biroi*	Barito Kuala	71.50 ± 0.42	39.30 ± 0.73
Jav_TB6	*T. biroi*	Magelang	80.34 ± 0.87	25.46 ± 0.84
Jav_TL1	*T. laeviceps*	Pandeglang	63.08 ± 0.68	44.56 ± 2.81
Jav_TL2	*T. laeviceps*	Batang	55.40 ± 0.93	45.04 ± 1.15
Jav_TL3	*T. laeviceps*	Magelang	33.08 ± 4.27	30.74 ± 3.95
Kal_TL4	*T. laeviceps*	Hulu Sungai Tengah	114.92 ± 15.26	74.40 ± 3.63
Kal_TL5	*T. laeviceps*	Barito Kuala	32.99 ± 0.66	20.66 ± 4.08
Kal_GT1	*G. thoracica*	Kutai Kartanegara	174.2 ± 12.09	94.18 ± 5.02
Sum_GT2	*G. thoracica*	Lebong	81.34 ± 2.61	49.58 ± 9.4
Kal_GT3	*G. thoracica*	Hulu Sungai Tengah	58.10 ± 4.89	43.52 ± 5.27
Sum_GT4	*G. thoracica*	Pematang Siantar	374.20 ± 0.51	194.18 ± 0.98
NT_SB1	*T. clypearis*	West Lombok	144.2 ± 0.75	81.02 ± 0.67
NT_SB2	*T. clypearis*	Central Lombok	134.86 ± 7.43	57.32 ± 1.61
Jav_SB3	*T. drescheri*	Batang	79.44 ± 0.42	43.5 ± 0.73

**Table 2:** Results of antioxidant activity using DPPH and ABTS methods.

Sample Name	IC_50_ DPPH (ppm)	IC_50_ ABTS (ppm)	Activity
Jav_HI1	65.4 ± 4.15	56.66 ± 9.64	Strong
Jav_HI2	57.78 ± 3.32	56.71 ± 0.47	Strong
Kal_HI3	28.87 ± 6.05	39.71 ± 0.79	Very strong
Kal_HI4	25.64 ± 0.55	38.79 ± 0.65	Very strong
Sum_HI5	59.45 ± 3.23	60.24 ± 3.69	Strong
NT_TB1	19.03 ± 0.08	26.34 ± 3.64	Very strong
Kal_TB2	47.73 ± 4.02	46.46 ± 5.61	Very strong
Jav_TB3	115.28 ± 2.30	117.21 ± 10.76	Moderate
Jav_TB4	70.30 ± 1.40	64.96 ± 4.43	Strong
Kal_TB5	61.69 ± 0.25	63.78 ± 0.29	Strong
Jav_TB6	50.79 ± 0.25	52.78 ± 0.45	Strong
Jav_TL1	116.82 ± 2.34	108.59 ± 1.97	Moderate
Jav_TL2	118.10 ± 2.36	110.21 ± 10.27	Moderate
Jav_TL3	83.37 ± 6.07	93.13 ± 4.00	Strong
Kal_TL4	70.03 ± 0.92	64.75 ± 1.84	Strong
Kal_TL5	83.16 ± 3.28	89.93 ± 1.77	Strong
Kal_GT1	46.20 ± 1.65	37.78 ± 1.42	Very strong
Sum_GT2	57.28 ± 0.48	67.35 ± 2.24	Strong
Kal_GT3	69.34 ± 5.13	68.29 ± 3.08	Strong
Sum_GT4	11.06 ± 0.55	17.29 ± 0.34	Very strong
NT_SB1	31.12 ± 0.99	45.14 ± 4.25	Very strong
NT_SB2	41.75 ± 0.96	43.63 ± 0.54	Very strong
Jav_SB3	51.69 ± 0.35	53.77 ± 0.9	Strong

**Table 3:** Grouping of stingless bee propolis from Indonesia.

Group	Sample	Species	Region	Island
Group 1	HI2	*H. itama*	Kendal	Java
	HI1	*H. itama*	Magelang	Java
	HI3	*H. itama*	Banjar Baru	Kalimantan
	HI4	*H. itama*	Kutai Kartanegara	Kalimantan
	HI5	*H. itama*	Kampar	Sumatra
Group 2	GT1	*G. thoracica*	Kutai Kartanegara	Kalimantan
	GT2	*G. thoracica*	Lebong	Sumatra
Group 3	TB2	*T. biroi*	Tanah Laut	Kalimantan
	TB3	*T. biroi*	Batang	Java
	TB4	*T. biroi*	Bogor	Java
	TB6	*T. biroi*	Magelang	Java
	TL1	*T. laeviceps*	Pandeglang	Java
	TL2	*T. laeviceps*	Batang	Java
	TL3	*T. laeviceps*	Magelang	Java
	TL4	*T. laeviceps*	Hulu Sungai Tengah	Kalimantan
	TL5	*T. laeviceps*	Barito Kuala	Kalimantan
	SB3	*T. drescheri*	Batang	Java
	GT3	*G. thoracica*	Hulu Sungai Tengah	Kalimantan
Group 4	TB1	*T. biroi*	Sumbawa	Nusa Tenggara
	TB5	*T. biroi*	Barito Kuala	Kalimantan
	SB1	*T. clypearis*	West Lombok	Nusa Tenggara
Group 5	SB2	*T. clypearis*	Central Lombok	Nusa Tenggara
	GT4	*G. thoracica*	Pematang Siantar	Sumatra

**Table 4:** Propolis compound results with FTIR.

Wave Number (cm^−1^)	Vibrational Modes	Position Band Sample				
		G1	G2	G3	G4	G5
3500–3200	O-H of alcohols, phenols, and carboxylic acids	-	√	√	-	√
3400–3250	N-H of amines	-	√	-	-	√
3000–2850	CH_3_, CH_2_, and CH of alkenes	√	√	√	√	√
1810–1640	C=O	√	√	√	√	√
1680–1600	C=C of conjugated aromatic rings	-	√	√	√	√
1550–1475	N-O	√	√	√	-	-
1500–1400	CH of aromatic rings	√	√	√	√	√
1470–1450	C-H	-	√	-	√	-
1360–1290	N-O	-	-	√	-	√
1335–1250	C-N	√	-	√	√	√
1320–1000	C-O of carboxylic acids, phenols, esters	√	√	√	√	√
1300–1150	CH_2_ of alkenes	√	√	√	√	√
1250–1020	C-N	√	√	√	√	√

**Table 5:** Compound of propolis Sum_GT4.

No.	Compound	RT (min)	Formula	Molecular Weight
1	Unknown compound	0.99	C_6_H_8_O_5_	161.0363
2	4-Methoxybenzaldehyde	7.76	C_8_H_8_O_2_	136.0524
3	Apocynin	8.52	C_9_H_10_O_3_	166.0630
4	Eupatoriochromene	9.12	C_13_H_14_O_3_	218.0943
5	Unknown compound	10.37	C_9_H_8_O	132.0575
6	(-)-8-Prenylnaringenin	11.84	C_20_H_20_O_5_	340.1311
7	trans-Cinnamaldehyde	12.47	C_9_H_8_O	132.0575
8	Glabridin	16.01	C_20_H_20_O_4_	324.1362
9	(1*R*,2*R*,5*S*,8*R*,10*R*,14*R*)-20-hydroxy-1,2,14,18,18-pentamethyl-17-oxo-8-(prop-1-en-2-yl) pentacyclohenicosane-5-carboxylic acid	17.86	C_30_H_46_O_4_	470.3396
10	Abietic acid	24.70	C_20_H_30_O_2_	302.2246

**Table 6:** List of potential marker compounds of propolis from *H. itama* and *T. laeviceps*.

No.	Compound Name	RT (min)	Formula	Molecular Weight	HI	TL
1	Petunidin	8.12	C_16_H_13_O_7_	318.0758	High	Low
2	Gingerone C	10.35	C_20_H_22_O_4_	327.1682	High	Low
3	L-β-aspartyl-L-leucine	10.97	C_10_H_18_N_2_O_5_	247.1304	High	Low
4	Flazine	10.67	C_17_H_12_N_2_O_4_	309.0859	High	Low
5	Pinusolide	18.93	C_21_H_30_O_4_	347.2195	Low	High
6	Prostaglandine F2a	12.93	C_20_H_34_O_5_	355.2441	High	Low
7	Mannitol 1-phosphate	1.51	C_6_H_15_O_9_P	263.0660	High	Low
8	Hydroxyprolyl-Isoleucine	1.59	C_11_H_20_N_2_O_4_	245.1441	Low	High
9	Cucurbic acid	9.23	C_12_H_20_O_3_	213.1611	High	Low
10	Galacticol	1.09	C_6_H_14_O_6_	183.0830	High	Low

**Table 7:** List of propolis samples.

No.	Sample Code	Location (City and Province)	Bee Species
1	Jav_HI1	Magelang, Central Java	*H. itama*
2	Jav_HI2	Kendal, Central Java	*H. itama*
3	Kal_HI3	Banjar Baru, South Kalimantan	*H. itama*
4	Kal_HI4	Kutai Kartanegara, East Kalimantan	*H. itama*
5	Sum_HI5	Kampar, Riau	*H. itama*
6	NT_TB1	Sumbawa, West Nusa Tenggara	*T. biroi*
7	Kal_TB2	Tanah Laut, South Kalimantan	*T. biroi*
8	Jav_TB3	Batang, Central Java	*T. biroi*
9	Jav_TB4	Bogor, West Java	*T. biroi*
10	Kal_TB5	Barito Kuala, South Kalimantan	*T. biroi*
11	Jav_TB6	Magelang, Central Java	*T. biroi*
12	Jav_TL1	Pandeglang, Banten	*T. laeviceps*
13	Jav_TL2	Batang, Central Java	*T. laeviceps*
14	Jav_TL3	Magelang, Central Java	*T. laeviceps*
15	Kal_TL4	Hulu Sungai Tengah, East Kalimantan	*T. laeviceps*
16	Kal_TL5	Barito Kuala, South Kalimantan	*T. laeviceps*
17	Kal_GT1	Kutai Kartanegara, East Kalimantan	*G. thoracica*
18	Sum_GT2	Lebong, Bengkulu	*G. thoracica*
19	Kal_GT3	Hulu Sungai Tengah, East Kalimantan	*G. thoracica*
20	Sum_GT4	Pematang Siantar, Nort Sumatra	*G. thoracica*
21	NT_SB1	West Lombok, West Nusa Tenggara	*T. clypearis*
22	NT_SB2	Central Lombok, West Nusa Tenggara	*T. clypearis*
23	Jav_SB3	Batang, Central Java	*T. drescheri*

**Table 8:** Procedure for testing antioxidant activity using the DPPH method.

Reagent	Volume (μL)		
	Blank	Control	Sample
EEP	-	-	20
DPPH 150 μmol/L	-	180	180
Methanol	200	20	-

Shaken for 60 s and incubated for 40 min at dark room. Absorbance was measured at λ 516 nm.

**Table 9:** Procedure for testing antioxidant activity using the ABTS method.

Reagent	Volume (μL)		
	Blank	Control	Sample
EEP	-	-	50
ABTS solution	-	50	50
Ethanol	100	50	-

Shaken for 60 s and incubated for 6 min. Absorbance was measured at λ 734 nm.

## Data Availability

Data are contained within the article.
